# *FANCM* and *RECQL* genetic variants and breast cancer susceptibility: relevance to South Poland and West Ukraine

**DOI:** 10.1186/s12881-018-0524-x

**Published:** 2018-01-19

**Authors:** Tú Nguyen-Dumont, Aleksander Myszka, Pawel Karpinski, Maria M. Sasiadek, Hayane Akopyan, Fleur Hammet, Helen Tsimiklis, Daniel J. Park, Bernard J. Pope, Ryszard Slezak, Nataliya Kitsera, Aleksandra Siekierzynska, Melissa C. Southey

**Affiliations:** 1 Genetic Epidemiology Laboratory, Department of Pathology, The University of Melbourne, Melbourne, Australia; 2 Institute of Obstetrics and Emergency Medicine, University of Rzeszow, Rzeszow, Poland; 3 Department of Genetics, Wroclaw Medical University, Wroclaw, Poland; 4 Institute of Hereditary Pathology of National Academy of Medical Sciences, Lviv, Ukraine; 5 Melbourne Bioinformatics, The University of Melbourne, Carlton, Victoria Australia; 6 Department of Biotechnology and Plant Physiology, University of Rzeszow, Rzeszow, Poland

**Keywords:** *FANCM*, *RECQL*, Breast cancer predisposition, Familial breast cancer, Gene panel testing

## Abstract

**Background:**

*FANCM* and *RECQL* have recently been reported as breast cancer susceptibility genes and it has been suggested that they should be included on gene panel tests for breast cancer predisposition. However, the clinical value of testing for mutations in *RECQL* and *FANCM* remains to be determined. In this study, we have characterised the spectrum of *FANCM* and *RECQL* mutations in women affected with breast or ovarian cancer from South-West Poland and West Ukraine.

**Methods:**

We applied Hi-Plex, an amplicon-based enrichment method for targeted massively parallel sequencing, to screen the coding exons and proximal intron-exon junctions of *FANCM* and *RECQL* in germline DNA from unrelated women affected with breast cancer (*n* = 338) and ovarian cancer (*n* = 89) from Poland (*n* = 304) and Ukraine (*n* = 123). These women were at high-risk of carrying a genetic predisposition to breast and/or ovarian cancer due to a family history and/or early-onset disease.

**Results:**

Among 427 women screened, we identified one carrier of the *FANCM:*c.1972C > T nonsense mutation (0.23%), and two carriers of the frameshift insertion *FANCM:*c.1491dup (0.47%). None of the variants we observed in *RECQL* were predicted to be loss-of-function mutations by standard variant effect prediction tools.

**Conclusions:**

Our study of the Polish and Ukrainian populations has identified a carrier frequency of truncating mutations in *FANCM* consistent with previous reports. Although initial reports suggesting that mutations in *RECQL* could be associated with increased breast cancer risk included women from Poland and identified the *RECQL:*c.1667_1667 + 3delAGTA mutation in 0.23–0.35% of breast cancer cases, we did not observe any carriers in our study cohort. Continued screening, both in research and diagnostic settings, will enable the accumulation of data that is needed to establish the clinical utility of including *RECQL* and *FANCM* on gene panel tests.

**Electronic supplementary material:**

The online version of this article (10.1186/s12881-018-0524-x) contains supplementary material, which is available to authorized users.

## Background

Genetic testing laboratories have adopted gene panel testing involving massively parallel sequencing to test for genetic susceptibility to breast and other cancers. A large number of genes can now be screened in single assay, at considerably reduced cost, thus providing opportunities for the large number of women who will undergo gene panel testing, including those who have never undergone testing and those previously not found to carry mutations in *BRCA1* and *BRCA2*. By increasing the diagnostic yield through the analysis of more breast cancer susceptibility genes, gene panel testing provides more women and their families the opportunity to receive personalised risk assessment and risk management. Given their potential to provide both a clinical service genetic test and accumulate important data related to rare genetic variants in a large number of candidate breast cancer susceptibility genes, panel tests often include more than the *bona fide* breast cancer predisposition genes [1, 2].

*FANCM* and *RECQL* are two such candidate breast cancer genes. As detailed below, both were identified as candidate genes through a whole-exome sequencing (WES) approach applied to multiple breast cancer families not known to carry pathogenic mutations in *BRCA1* and *BRCA2.* Further evidence was subsequently sought using case-control analyses (summarized in Additional file 1: Table S1). These genes have already been included in some of the commercially available gene panel tests for breast cancer susceptibility, for instance the BROCA Cancer Risk Panel [3].

FANCM is a member of the Fanconi anemia complemetation group (FANC). The mutations *FANCM*:c.5701C > T; p.Gln1701* and *FANCM*:c.5791C > T; p.Arg1931* were identified by Kiiski et al. [4] and Peterlongo et al. [5], respectively. Kiiski et al. followed-up on their WES findings by genotyping and found 96/3079 (3.1%) women affected with breast cancer and 38/2080 (1.5%) controls carrying the mutation (Odds Ratio (OR) = 1.86; 95% CI [1.26–2.75]; *p* = 0.0018). For cases with a family history, the reported OR was 2.11; 95% CI [1.34–3.32]; *p* = 0.0012 [4]. Peterlongo et al. genotyped *FANCM*:c.5791C > T in 8635 familial breast cancer cases and 6625 controls from Italy, France, Spain, Germany, Australia, USA, Sweden and The Netherlands. The mutation was identified in 18/8635 (0.21%) breast cancer cases and in 4/6625 (0.06%) controls (OR = 3.93; 95% CI [1.28–12.11]; *p* = 0.017) [5]. A case-control screening of the coding region of *FANCM* was recently performed in 2047 familial breast cancer cases, 628 ovarian cancer cases and 2187 controls from Germany [6]. The authors focused on *FANCM* loss-of-function mutations and reported an OR of 2.44; 95% CI [1.08–5.59]; *p* = 0.02 for breast cancer cases diagnosed before the age of 51.

RECQL is a DNA helicase of the RecQ family implicated in the maintenance of genome stability. In a study of the Northern Chinese population, Sun et al. identified 9/448 breast cancer affected women carrying deleterious mutations in *RECQL*, as compared to 1/1588 unaffected women (*p* < 0.001) [7]. Through WES of 195 Polish and French-Canadian women affected by breast cancer, Cybulski et al. also identified *RECQL* as a plausible candidate gene for breast cancer susceptibility [8]. Large scale validation was undertaken for two mutations. The first mutation, *RECQL:*c.643C > T;p.Arg215*, was observed in 7/1013 (0.69%) French-Canadian affected women and 1/7136 (0.014%) controls (*p* < 0.001). The second mutation, *RECQL:*c.1667_1667 + 3delAGTA, was genotyped in 13,136 unselected Polish women with breast cancer and 4702 cancer-free Polish controls. The mutation was observed in 30 cases (0.23%) and 2 controls (0.04%) (OR =5.4; 95% CI [1.3–46]; *p* = 0.008).

Following the discovery of these “new breast cancer genes”, it has been suggested that *RECQL* and *FANCM* should be included on gene panel tests for breast cancer susceptibility [6, 9]. However, the clinical value of testing for mutations in *RECQL* and *FANCM* remains to be determined. For instance, *RECQL:*c.1667_1667 + 3delAGTA has been further investigated in a recent case-control study from Belarus and Germany, which comprised 2596 breast cancer affected women and 2132 unaffected women [10]. In their study, Bogdanova et al. identified the mutation in nine breast cancer cases (0.35%) and six controls (0.28%), with an OR = 1.23; 95% CI [0.44–3.47]; *p* = 0.69. A meta-analysis comprising the initial study of Cybulski et al. yielded an OR = 2.51; 95%CI [1.13–5.57]; *p* = 0.02, thus suggesting that *RECQL:*c.1667_1667 + 3delAGTA could be a moderate-risk, rather than a high-risk mutation for breast cancer susceptibility.

These results suggest that additional studies are warranted to establish the contribution of mutations in these genes to breast cancer susceptibility. Here we report the application of massively parallel sequencing to characterise the germline mutation spectrum of *RECQL* and *FANCM* in unselected women from South-West Poland and West Ukraine affected with breast or ovarian cancer.

## Methods

### Study participants

Participants in this study were unrelated women diagnosed with breast or ovarian cancer recruited after or during the oncological treatment from Wroclaw Medical University, Lower Silesia, Poland, between 2004 and 2008, or Lviv State Oncology Regional Treatment and Diagnostic Center, Lviv, Ukraine between 2004 and 2010 (Table 1). Genetic testing was requested when hereditary cancer was suspected (age of onset < 50, bilateral breast cancer, medullary or atypical breast cancer, more than one breast cancer in the family occurring in a first or second degree relative and ovarian cancer in any age). The time from cancer diagnosis to blood draw ranged from one to 12 months.

**Table 1:** Characteristics of the participants to this study

	Breast cancer		Ovarian cancer	
	Poland (*n* = 226)	Ukraine (*n* = 112)	Poland (*n* = 78)	Ukraine (*n* = 11)
Age at diagnosis (years)	49 (22–72)	50 (28–79)	53 (25–80)	51 (31–65)
Relatives with breast cancer:				
0	148 (65%)	24 (21%)	61 (%)	5 (45%)
1	51 (23%)	56 (50%)	14 (%)	6 (55%)
2+	27 (12%)	32 (29%)	3 (%)	0 (0%)
Relatives with ovarian cancer:				
0	210 (93%)	98 (86%)	65 (%)	6 (55%)
1	13 (6%)	12 (11%)	10 (%)	3 (27%)
2+	3 (1%)	2 (2%)	3 (%)	2 (18%)
Invasive cancers grading				
GI	14 (11%)	NA	8 (18%)	NA
GII	65 (52%)	NA	18 (41%)	NA
GIII	46 (37%)	NA	18 (41%)	NA
In situ cancers grading				
GI	1 (20%)	NA	–	–
GII	2 (40%)	NA	–	–
GIII	2 (40%)	NA	–	–

*NA: data not available

GI, grade I; GII, grade II; GIII, Grade III

The Polish cohort consisted of 226 women affected with breast cancer and 78 women affected with ovarian cancer. Of the 226 women with breast cancer, 85 had hereditary breast cancer, 17 had familial breast cancer and 124 were sporadic cases, according to the criteria described by Berliner et al. [11]. The majority of these women (*n* = 206, 91%) had been diagnosed with invasive cancer (ductal in 153, lobular in 30, medullary in 7, tubular in 5 cases, 11 patients have been diagnosed with other types of carcinoma). There were 20 cases of cancer in situ (ductal carcinoma in situ in 19, lobular carcinoma in situ in 1). Of the 78 Polish women with ovarian cancer, 11 had hereditary ovarian cancer, 10 had familial ovarian cancer and 57 were sporadic ovarian cancer cases. Of these ovarian cancers, 38 were serous, 15 were endometroid, 10 were mucinous, two were clear cell carcinomas and 13 were adenocarcinomas not otherwise specified. Known carriers of Polish founder mutations in *BRCA1* (c.5266dup, c.181 T > G, c.4035del, c.68_69del) and in *BRCA2* (c.5946delT) were excluded from this study.

The Ukrainian cohort consisted of 112 women with breast cancer and 11 women with ovarian cancer. Seventy-four women affected with breast cancer were diagnosed with hereditary cancer and 38 with familial cancer. There were 78, 18 and 2 cases of invasive ductal, lobular and medullary breast cancers, respectively. Of the 11 Ukrainian women with ovarian cancer, two had moderately differentiated carcinoma, one had endometroid adenocarcinoma, one had serous papillary adenocarcinoma, one had low-grade differentiated adenocarcinoma and six had adenocarcinoma not otherwise specified.

### Ethics, consent and permissions

All participants provided informed consent for participation in this research program, which was approved by the Commission of Bioethics of the Institute of Hereditary Pathology of the National Academy of Medical Sciences of Ukraine, the Ethics Committee of Wroclaw Medical University (Poland), the Ethics Committee of University of Rzeszow (Poland) and the University of Melbourne Human Research Ethics Committee (Melbourne, Australia).

### Mutation screening

Amplicon-based massively parallel sequencing of the coding regions and proximal intron-exon junctions of *FANCM* (NM_020937.3) and *RECQL* (NM_002907.3) was performed on lymphocytes-derived germline DNA using the Hi-Plex protocol [12]. Massively parallel sequencing (150 bp paired-end) was performed on the MiSeq (Illumina, San Diego, CA, USA). Mapping to the human reference build hg19 and variant calling were performed as described in [12, 13].

### In-silico analysis

DNA sequence variant annotation (variant nomenclature and type, and dbSNP138 identifier) was performed using CAVA [14]. The probability that missense substitutions in *FANCM* or *RECQL* were damaging to protein function was assessed with PolyPhen-2 [15] and CADD [16]. The threshold for calling a missense variant damaging was the default for PolyPhen-2. The cutoff for CADD was 15, as recommended by the authors. Minor Allele Frequency (MAF) was obtained for non-Finnish European ancestry from the ExAC database [17].

## Results

### FANCM

A total of 31 distinct *FANCM* genetic variants were observed (Table 2). We identified one carrier of the nonsense mutation: *FANCM:*c.1972C > T;p.(Arg658*) and two carriers of a frameshift mutation resulting in a predicted premature termination codon: *FANCM:*c.1491dup; p.(Ser498fs). Of the 24 missense variants identified in the mutation screening, three were predicted probably damaging by an *in-silico* analysis using Polyphen-2 and CADD. The remaining variants were synonymous variants. No variant affecting consensus splice sites was detected. No ovarian cancer cases were found to carry a loss-of-function mutation in *FANCM*.

**Table 2:** *FANCM* variants identified by Hi-Plex targeted-sequencing, in 427 women affected with breast or ovarian cancer in South-West Poland and West Ukraine

	HGVS_c^a^	HGVS_p^a^	dbSNP^b^	MAF in ExAC^c^	# Carriers	PolyPhen-2	CADD
Nonsense mutation	c.1972C > T	p.Arg658*	rs368728266	0.0001	1	.	.
Frameshifting mutation	c.1491dup	p.Gln498Thrfs*7	.	4.594e-05	2	.	.
Missense substitutions	c.229A > G c.524C > T c.527C > T c.624A > G c.1576C > G c.1964A > G c.2632G > T c.2859A > C c.3173A > G c.3296G > A c.3407 T > C c.3758A > G c.3902A > T c.3935 T > C c.4184G > T c.4378A > G c.4465G > A c.4627C > T c.4799C > T c.4934G > A c.5003 T > G c.5224A > G c.5434C > G c.5627A > G	p.T77A p.S175F p.T176I p.I208M p.L526 V p.N655S p.V878 L p.K953 N p.N1058S p.R1099H p.L1136S p.N1253S p.D1301V p.L1312P p.G1395 V p.I1460V p.G1489R p.L1543F p.T1600I p.R1645H p.I1668S p.I1742V p.P1812A p.N1876S	rs61746895 rs10138997 rs77374493 rs45547534 rs144215747 rs61753893 rs1367580 rs142864437 . rs139382267 . rs45604036 . rs200028975 . rs78211950 rs183784665 rs139536545 rs61746943 . . rs143662421 rs3736772 rs45557033	0.013 0.062 0.0056 0.015 0.0011 0.01 0.12 0.0018 0 0.0003 0 0.03 . 0.0001 . 0.11 . 0.0005 0.027 0 . 0.012 0.11 0.029	6 22 2 6 2 6 47 1 1 1 1 20 1 1 1 7 1 2 19 1 1 8 43 20	B B B D B B B D B B D B B B B B B D P B D B B B	2.724 10.68 10.31 18.2 13.05 11.72 0 14.93 0.053 0.003 15.54 0.52 7.048 8.403 1.149 0.01 2.925 11.61 16.1 9.018 20.8 0.102 9.077 8.271
Synonymous substitutions	c.459 T > C c.3309C > T c.4161 T > C c.5598G > A c.6141 T > C	p.A153A p.H1103H p.S1387S p.R1866R p.D2047D	.	.	1	.	.
			.	.	1	.	.
			.	.	2	.	.
			.	.	1	.	.
			rs8018014	0.013	9	.	.

^a^Variant nomenclature based on transcript sequence (NM_020937.3), + 1 as A of ATG start codon, according to the Human Genome Variation Society (HGVS), HGVS_c for coding DNA and HGVS_p for protein variants

^b^dbSNP 138

^c^Minor Allele Frequency (MAF) in ExAC Non-Finnish European population [17]

The woman carrying the nonsense mutation *FANCM:*c.1972C > T was diagnosed with invasive ductal carcinoma at the age of 62. Other cancers in the paternal lineage included lung cancer in her father, urinary tract cancer in her uncle and breast cancer in a first-degree cousin. Her maternal aunt had pancreatic cancer.

**Fig. 1:**
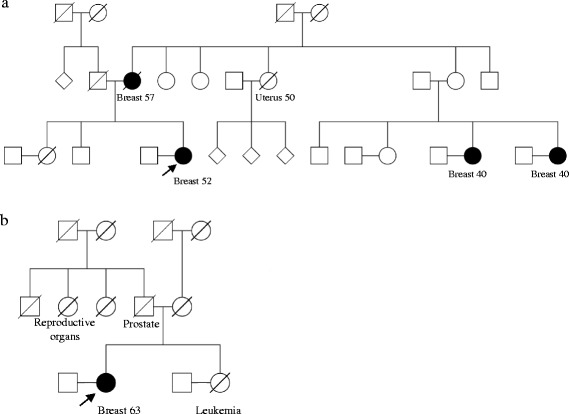
**a** and **b**: Pedigree of the families carrying *FANCM:*c.1491dup; p.(Ser498fs).The arrow indicates the study participants found to carry *FANCM*:c.1491dup. Breast cancer is indicated by black filled symbols. Age at diagnosis and other cancers are indicated when known

*FANCM:*c.1491dup was observed in two women with breast cancer. Their family pedigrees are reported in Fig. 1. The first woman was diagnosed with invasive ductal carcinoma at the age of 52. Her mother had been diagnosed with breast cancer (age at diagnosis, dx: 57 years old) and her aunt had been diagnosed with cancer of the uterus (dx: 50 years old). Two of her maternal cousins were diagnosed with breast cancer at the age of 40 (Fig. 1-a). The second woman carrying *FANCM:*c.1491dup was diagnosed with invasive ductal carcinoma at the age of 63. Other cancers in her family included prostate cancer in her father, reproductive organs cancer in her paternal aunt and leukemia in her sister (Fig. 1-b).

### RECQL

A total of nine distinct *RECQL* genetic variants were observed in our cohort of 427 women affected by breast or ovarian cancer. However, none of those were predicted loss-of-function mutations. Of the five missense substitution identified, *RECQL*: c.386G > A; p.Cys129Tyr was the only predicted to be damaging by Polyphen-2 and CADD. There were five synonymous variants (Table 3).

**Table 3:** *RECQL* variants identified by Hi-Plex targeted-sequencing, in 427 women affected with breast or ovarian cancer in South-West Poland and West Ukraine

	HGVS_c^a^	HGVS_p^a^	dbSNP^b^	ExAC^c^	Polyphen-2^d^	CADD^e^	# Carriers
Missense substitutions	c.156 T > G	p.Asp52Glu	.	0.00002	0.001,B	5.347	1
	c.386G > A	p.Cys129Tyr	rs187203579	0.00009	0.914,D	22.5	1
	c.1483G > C	p.Asp495His	rs6499	0.00520	0.05, B	16.05	4
	c.1743 T > A	p.Asn581Lys	.	.	0.009,B	12.03	2
Synonymous substitutions	c.87G > A	p.Thr29Thr	.	0.00005			1
	c.393 T > C	p.Asp131Asp		.			1
	c.1536A > T	p.Pro512Pro	.	0.00005			1
	c.1731 T > C	p.Asn577Asn	rs6500	0.08850			67
	c.1899A > G	p.Gln633Gln	rs61754415	0.08832			65

^a^Variant nomenclature based on transcript sequence (NM_002907.3), + 1 as A of ATG start codon, according to the Human Genome Variation Society (HGVS), HGVS_c for coding DNA and HGVS_p for protein variants

^b^dbSNP 138

^c^Minor Allele Frequency (MAF) in ExAC Non-Finnish European population [17]

^d^PolyPhen-2 prediction: B, benign; D: damaging [15]

^e^CADD phred-scaled score [16]

## Discussion

The present study of breast and ovarian cancer cases from Poland and Ukraine identified 1/427 (0.23%) carrier of the truncating *FANCM:*c.1972C > T mutation. This is consistent with previously reported carrier frequency in early-onset breast cancer cases. *FANCM:*c.1972C > T had been observed in 4/2047 (0.20%) familial breast cancer cases by Neidhardt et al. in their study of the German population. The carrier frequency of this nonsense mutation in ExAC is 0.01% in the non-Finnish European population. We identified 2/427 (0.47%) carriers of the frameshift insertion *FANCM:*c.1491dup. This mutation was not observed by Neidhart et al. but is present in the ExAC database at a carrier frequency of 0.008%. There are currently no published reports of missense substitutions in *FANCM* leading to disruption of FANCM protein function.

Our study of the Polish and Ukrainian populations has identified carrier frequency of truncating mutations in *FANCM* consistent with previous reports. Although initial reports suggesting that mutations in *RECQL* could be associated with increased breast cancer risk included women from Poland and identified the *RECQL:*c.1667_1667 + 3delAGTA mutation in 0.23% [8] to 0.35% [10] of breast cancer cases, we did not identify any carriers in this study cohort.

## Conclusions

In order to estimate individual breast cancer risk for carriers of mutations in *RECQL* and *FANCM*, very large sample sizes and family based studies are required*.* With the inclusion of these genes in panel tests for breast cancer susceptibility, both in research and clinical settings, additional families with mutations will be identified and contribute to a better understanding of the breast cancer risk associated with mutations in these genes*.* Although interpretation of the genetic data derived from this testing will be limited in the short term, data will accumulate, ultimately enabling a better understanding of the role of *RECQL* and *FANCM* in susceptibility to breast cancer and establishing the clinical utility of including these genes on gene panel tests. This data could also contribute to work assessing the possible association between ovarian cancer risk and *RECQL* and *FANCM* mutations for which there is currently no evidence.

## Additional file

Additional file 1: Table S1. *FANCM* and *RECQL* truncating mutations characterized by case-control analyses, following their initial involvement in breast cancer susceptibility via a whole-exome sequencing approach. List of *FANCM* and *RECQL* truncation mutations identified by case-control analyses and corresponding references. (DOCX 19 kb)

## Abbreviations

*BRCA1*: BRCA1, DNA repair associated
*BRCA2*: *BRCA2*, DNA repair associated
ExAC: Exome Aggregation Consortium
*FANCM*: Fanconi Anemia Complementation Group M
HGVS: Human Genome Variation Society;
MAF: Minor Allele Frequency
*RECQL*: RecQ Like Helicase
