# Supplementary material for: FANCM and RECQL genetic variants and breast cancer susceptibility: relevance to South Poland and West Ukraine
Source: BMC Med Genet. 2018 Jan 19;19:12. doi: 10.1186/s12881-018-0524-x (PMC5775547; doi:10.1186/s12881-018-0524-x)
Supplement: Additional file 1: Table S1. — FANCM and RECQL truncating mutations characterized by case-control analyses, following their initial involvement in breast cancer susceptibility via a whole-exome sequencing approach. List of FANCM and RECQL truncation mutations identified by case-control analyses and corresponding references. (DOCX 19 kb) [file 12881_2018_524_MOESM1_ESM.docx]

| **Gene** | **Mutation** | **Case-carrier frequency**  **(%)** | **Control-carrier frequency**  **(%)** | **OR**  **[95% CI]** | **p-value** | **Population** | **References** |
| --- | --- | --- | --- | --- | --- | --- | --- |
| *FANCM* | c.5701C>T; p.Gln1701* | 96/3,079  (3.1) | 96/3,079  (3.1) | 1.86  [1.26-2.75] | 0.0018 | Finland | Kiiski et al [4] |
|  | c.5791C>T; p.Arg1931* | 18/8,635 (0.21) | 4/6,625  (0.06) | 3.93  [1.28-12.11] | 0.017 | Europe, Australia, USA | Peterlongo et al [5] |
| *RECQL* | c.643C>T;  p.Arg215*, | 7/1,013  (0.69) | 1/7,136  (0.014) | NA | <0.001 | French Canadian | Cybulski et al [8] |
|  | c.1667_1667+3delAGTA | 30/13,136 (0.23) | 2/4,702 (0.04) | 5.4  [1.36-46] | 0.008 | Polish | Cybulski et al [8] |
